# Recent Advances in Nanoparticle and Nanocomposite-Based Photodynamic Therapy for Cervical Cancer: A Review

**DOI:** 10.3390/cancers17152572

**Published:** 2025-08-04

**Authors:** Dorota Bartusik-Aebisher, Mohammad A. Saad, Agnieszka Przygórzewska, David Aebisher

**Affiliations:** 1 Department of Biochemistry and General Chemistry, College of Medicine, Faculty of Medicine, University of Rzeszów, 35-310 Rzeszów, Poland; dbartusikaebisher@ur.edu.pl; 2 Wellman Center for Photomedicine, Massachusetts General Hospital and Harvard Medical School, Boston, MA 02114, USA; msaad1@mgh.harvard.edu; 3 English Division Students Science Club, College of Medicine, Faculty of Medicine, University of Rzeszów, 35-310 Rzeszów, Poland; ap117623@stud.ur.edu.pl; 4 Department of Photomedicine and Physical Chemistry, College of Medicine, Faculty of Medicine, University of Rzeszów, 35-310 Rzeszów, Poland

**Keywords:** photodynamic therapy, PDT, cervical cancer, nanoparticles, nanocomposites, cancer treatment

## Abstract

Cervical cancer represents a significant global health challenge. Photodynamic therapy (PDT) appears to be a promising, minimally invasive alternative to standard treatments. However, the clinical efficacy of PDT is sometimes limited by the low solubility and aggregation of photosensitizers, their non-selective distribution in the body, hypoxia in the tumor microenvironment, and limited light penetration. Recent advances in nanoparticle and nanocomposite platforms have addressed these challenges by integrating multiple functional components into a single delivery system. By encapsulating or conjugating photosensitizers in biodegradable matrices, such as mesoporous silica, organometallic structures and core–shell construct nanocarriers increase stability in water and extend circulation time, enabling both passive and active targeting through ligand decoration. Up-conversion and dual-wavelength responsive cores facilitate deep light conversion in tissues, while simultaneous delivery of hypoxia-modulating agents alleviates oxygen deprivation to sustain reactive oxygen species generation. Controllable “motor-cargo” constructs and surface modifications improve intratumoral diffusion, while aggregation-induced emission dyes and plasmonic elements support real-time imaging and quantitative monitoring of therapeutic response. Together, these multifunctional nanosystems have demonstrated potent cytotoxicity in vitro and significant tumor suppression in vivo in mouse models of cervical cancer. Combining targeted delivery, controlled release, hypoxia mitigation, and image guidance, engineered nanoparticles provide a versatile and powerful platform to overcome the current limitations of PDT and pave the way toward more effective, patient-specific treatments for cervical malignancies. Our review of the literature summarizes studies on nanoparticles and nanocomposites used in PDT monotherapy for cervical cancer, published between 2023 and July 2025.

## Simple Summary

Research on nanoparticles and nanocomposites used in photodynamic therapy aims to optimize this light-activated treatment for cervical cancer, which is less invasive than surgery or radiation therapy. Embedding light-sensitive drugs in nanoparticles and nanocomposites enhances their blood circulation time and promotes preferential accumulation in tumors, reducing side effects. Some designs convert invisible light into cancer-killing wavelengths deep in the tissue, while others incorporate oxygen-enhancing agents to help treatments work better in low-oxygen areas of tumors. Many also have built-in features that allow researchers and physicians to track in real time particle trafficking and their therapeutic efficacy. Together, these advances promise a more precise and effective approach to treating cervical cancer and could guide the development of similar therapies for other hard-to-treat cancers.

## 1. Introduction

Cervical cancer is one of the most common malignancies, ranking fourth in terms of incidence and mortality among women worldwide [1]. It is estimated that half of the women who have died from this cancer are under the age of 58 years, while in the 20–39 age group, cervical cancer is the second most common cause of cancer deaths [2]. Given its high incidence and significant health burden, it is becoming important to understand the molecular mechanisms underlying the development and progression of this cancer. A key initiating factor in carcinogenesis is chronic human papillomavirus (HPV) infection, especially high-risk types such as HPV16 and HPV18 [3]. Viral oncoproteins E6 and E7 lead to inactivation of the tumor suppressor proteins p53 and pRb, resulting in uncontrolled cell division, genome instability, and tumor progression [4]. Based on data from the TCGA database, molecular subtypes of cervical cancer were identified, including squamous cell carcinomas with different keratin content and an endometrial-like subtype, usually negative for HPV, which is characterized by frequent mutations in the KRAS, ARID1A, and PTEN genes [5,6]. The genes most commonly altered in this cancer also include PIK3CA, MAPK1, ERBB3, CASP8, and TGFBR2. A key role in progression is played by activation of the PI3K/Akt/mTOR pathway, exacerbated by the action of HPV oncoproteins, which promotes proliferation, migration, and resistance to treatment [6,7]. Other molecular pathways are also important in the pathogenesis of cervical cancer. The RAS/RAF/MAPK pathway promotes cell proliferation and differentiation, and its activation, including by KRAS mutations, is associated with a more aggressive course of the disease [8,9]. The Wnt/β-catenin pathway, through disruption of elements such as APC or CTNNB1, promotes the epithelial–mesenchymal transition (EMT) and tumor invasiveness [10]. The Notch and Hedgehog pathways are involved in the maintenance of cancer stem cells and promote metastasis. The NF-κB pathway, on the other hand, promotes cancer cell survival and the development of inflammation in the tumor microenvironment [11]. Additional mechanisms of progression include tumor hypoxia and overexpression of HIF-1α, CA9, and VEGF, which increase tumor aggressiveness [7]. Mutations in KRAS and overexpression of COX-2 and EGFR are associated with a poor prognosis and resistance to chemoradiotherapy [12,13]. Furthermore, abnormalities in DNA repair mechanisms, such as reduced levels of ATM, PARP-1, and Ku86 proteins, are associated with shorter survival and may indicate a potential benefit of PARP inhibitor therapy [14]. Epigenetic and post-transcriptional regulation plays an important role in the development of cervical cancer—noncoding RNA molecules such as miRNAs, lncRNAs, and circRNAs modulate pathways related to the cell cycle, EMT, and treatment response. Additionally, overexpression of some of these correlates with tumor progression and adverse prognosis [15,16]. Despite significant advances in understanding of the molecular basis of cervical cancer, as well as the development of prevention, diagnosis, and treatment methods, this disease still poses a major challenge to health systems and remains one of the leading causes of death among women [17]. Therefore, it is necessary to develop more precise and effective therapeutic strategies that could improve the prognosis of patients and reduce the mortality associated with this cancer.

Photodynamic therapy (PDT) has emerged in recent decades as an alternative, non-invasive therapeutic approach for the treatment of various diseases, including cancer, offering significant advantages over conventional approaches such as chemotherapy and radiation therapy [18]. Unlike these methods, PDT has higher selectivity of action, resulting in reduced toxic side effects and minimal damage to healthy tissues [19]. The basic mechanism of action of PDT is based on the interaction of three key elements: light, photosensitizer, and molecular oxygen [20]. The effectiveness of therapy depends on proper distribution of the photosensitizer and precise irradiation of the affected tissues with light of the appropriate wavelength. The process of irradiation leads to excitation of the photosensitizer and its transition through successive energy states, which allows energy transfer and the generation of reactive oxygen species (ROS) within the tumor [21,22]. The resulting ROS initiate a number of biological processes, such as direct cytotoxicity against cancer cells by inducing their apoptosis or necrosis [23], destroying tumor blood vessels as a result of the anti-angiogenesis effect, and stimulating the host immune system, enabling effective recognition and elimination of remaining cancer cells [24]. However, the effectiveness of PDT is limited by a number of challenges. Traditional photosensitizers are characterized by hydrophobicity, a tendency to aggregate, low biodistribution, and a lack of selectivity towards cancer cells, which negatively affects their photophysical and biological properties, significantly reducing the effectiveness of therapy [25,26,27]. Ideal photosensitizer delivery systems should be biocompatible, and degradable in the tumor microenvironment, and exhibit limited uptake by healthy cells [28]. Furthermore, one of the main challenges that limit the effectiveness of PDT remains hypoxia in solid tumors, which reduces the production of reactive oxygen species [29,30].

Nanoparticles, materials of 1 to 100 nm in size [31], and nanocomposites, i.e., composites containing nanometric components dispersed in a macroscopic matrix [32], represent a promising platform to overcome these limitations, acting as photosensitizer carriers to increase both the selectivity of delivery and the bioavailability of therapeutic molecules [33,34]. In addition to improving solubility, they also enable controlled release of drugs [35,36]. In passive targeting, nanoparticles and nanocomposites accumulate in the tumor as a result of the EPR effect [37], while active targeting involves functionalizing the surface of nanoparticles and nanocomposites with ligands that recognize specific tumor receptors, such as antibodies, their fragments, aptamers, or peptides [38,39,40,41,42]. Significantly, a single nanoparticle or nanocomposite can be functionalized with multiple copies of a single ligand or a combination of different ligands, further increasing the efficiency and specificity against cancer cells [43]. Numerous reports in the literature show promising results from the use of nanoparticles and nanocomposites in PDT, both as monotherapy and in combination with other therapeutic modalities [44,45,46]. With their surface conjugation capabilities and favorable physicochemical properties, nanoparticles and nanocomposites remain one of the most promising platforms in providing photosensitizers and supporting cancer treatment, including cervical cancer.

Our narrative review summarizes research on nanoparticles and nanocomposites for PDT monotherapy of cervical cancer published from 2023 to July 2025. An article search was conducted in the PubMed/MEDLINE, Scopus, and Web of Science database on 23 July 2025 using the phrase “PDT AND (nanoparticle* OR nanocomposite*)”. The inclusion and exclusion criteria of the articles retrieved for this review are presented in Table 1. Finally, 16 original scientific articles describing nanoparticles and nanocomposites in photodynamic therapy for cervical cancer qualified for inclusion.

## 2. Architecture of Nanoparticles and Nanocomposites in the PDT of Cervical Cancer

The structure of nanoparticles and nanocomposites plays an important role in the effectiveness of PDT. Properly designed architecture affects the stability of the photosensitizer, its solubility, and the efficiency of ROS generation [47,48]. A variety of structure types have been reported in the literature, including core–shell nanoparticles, polymeric systems, metallic hybrids, and porous nanocarriers, each offering unique functional properties. A review of selected publications on PDT of cervical cancer reveals a variety of structural approaches, among which nucleus–mantle structures and hybrid and polymer nanocomposites predominate.

One of the most frequently chosen design solutions was core–shell structures. These structures are a unique combination of two materials, using synergistic effects to integrate the unique attributes of both components [49]. The ability of a core–shell nanosystem to encapsulate and stabilize active substances is based on two key features: one or more cores (usually hydrophobic) and a hydrophilic outer surface (usually hydrophilic) [50]. Furthermore, due to the possibility of modifying both the core and the shell, these structures are highly flexible in design and can be adapted to specific therapeutic needs. Examples of such designs include a wide range of cores and shells, from upconverting NPs and silica to aggregation-induced emission (AIE) photosensitizer aggregates and cellular shells. The most commonly used cores were various UCNPs [51,52,53,54]. UCNPs are a class of luminescent nanomaterials capable of emitting light at wavelengths shorter than the exciting light [55]. This allows the conversion of longer wavelength light, which penetrates tissue better [56], into shorter-wavelength light suitable for activating the photosensitizer. This phenomenon is based on the anti-Stokes process and is referred to as photon upconversion, which involves the sequential absorption of two or more low-energy photons to fill actual indirectly excited electronic states, followed by the emission of a single high-energy photon [55]. All the analyzed studies confirmed the effective ability of UCNPs to absorb light in the near-infrared (NIR) range and emit light at a wavelength that allows activation of the photosensitizer [51,52,53,54]. Another type of core used in nanocomposites was photosensitizer molecules. Zhao et al. used AIE-photosensitizer MEO-TTMN aggregates [57]. Liu et al. developed a nanocomposite with cores composed of modified red phosphorus nanoparticles of graphitic carbon nitride—C_3_N_4_-RP [58]. The characteristics and properties of the photosensitizers used are discussed below. As the core in their nanostructure, Bai et al. used a porous metal–organic framework based on porphyrin, combining the core–shell approach with the characteristic of metal–organic framework (MOF) structures; a detailed description of the characteristics of MOFs can be found later in this chapter [59]. A wide variety of coatings have been used in core–shell nanosystems. Lin et al. used mesoporous silicon oxide [51]. Silicon oxide with a pore structure of 2–50 nm has a large specific surface area, adjustable particle size, and good biocompatibility [60], which results in high load capacity, controlled drug release, and the ability to modify the surface for targeted delivery and bioimaging [61]. Because of these properties, mesoporous silicon oxide is widely used as a nanocarrier in PDT and other forms of therapy. Mesoporous silicon oxide was also used by Wang et al., who developed an asymmetric coating with participation of both a photosensitizer and black Roussin salt [52]. Roussin’s black salt is a photolabile complex that releases nitric oxide (NO) when exposed to radiation [62].

Its incorporation into an asymmetric nanoparticle shell enables the generation of propulsive force, resulting in the directional movement of particles. This concept was confirmed by demonstrating that nanoparticles without Roussin’s black salt moved randomly, while UM-RZ nanoparticles (containing Roussin’s black salt) exhibited directional movement with a maximum speed of 194 µm·s^−1^ [52]. Zhao et al. used silicon oxide as a coating material, providing the nanoparticles with high dispersibility and structural stability [57], and pluronic F-127, an amphiphilic polymer approved by the FDA [63]—which also served as a template for the MEO-TTMN photosensitizer [57]. Nsubuga et al. used a layer of NaGdF_4_, which could support the stability of the upconversion luminescence and the efficiency of energy transfer to organic photosensitizers anchored in the phospholipid layer, translating into the effective production of singlet oxygen [53]. Liu et al. used a red blood cell membrane coating as a shell [58]. The red blood cell membrane is used as a nanoplatform coating due to its high biocompatibility, degradability, and exceptional deformability, making it an optimal therapeutic carrier [64]. Nanoplatforms coated with erythrocyte membranes show a significant increase in the efficacy of PDT [65] due to the effective delivery of photosensitizers to tumor sites and their specific targeting of tumor tissue [66]. Encapsulation of nanoparticles with red blood cell membranes is a simple and effective strategy to increase tumor accumulation and elimination [67]. A study by Liu et al. confirmed that, due to the presence of a cell membrane envelope on its surface, C_3_N_4_-RP@RBCm nanocomposites exhibit the advantages of high biocompatibility and cell uptake [58]. Chen et al. created a coating from a porphyrin MOF that acts as a photosensitizer, combining the advantages of a core–shell nanosystem and an MOF structure, the properties of which are discussed below. The MOF structure allowed for the simultaneous deposition of platinum nanoparticles (PtNPs) [54]. As coating, Bai et al. used a layer of hyaluronan applied to the surface of the porphyrin MOF by means of an electrostatic reaction [59]. As a natural polysaccharide [68], hyaluronic acid is characterized by high hydrophilicity, excellent biocompatibility, and nontoxicity [69,70,71]. Hyaluronic acid can also be degraded by enzymes that are overexpressed in the tumor environment, leading to the release of the therapeutic payload [69]. Furthermore, hyaluronic acid enables the targeting of nanosystems to the CD44 receptor [72], as described in the section on nanosystem delivery.

An alternative design developed to improve the functional properties of nanoplatforms is a structure that extends the classic core–shell arrangement to include additional layers, as in the case of core–shell–shell or core–shell–shell–shell architectures. These nanoplatforms consist of a central core and successive concentric layers of coatings, allowing precise control of their properties [73,74]. In both studies dedicated to PDT of cervical cancer, the UCNPs characterized above were used as the core. In the study by Ling et al., mesoporous silicon oxide was used as the first layer, while the second shell consisted of the photosensitizer BODIPY and the peptide FFYp, which promotes the internalization of nanosystems [75]. In the second study, which used a core–shell–shell–shell structure, Ling et al. used NaGdF_4_ surrounded by layers of silicon oxide and titanium oxide (TiO_2_) [76]. NaGdF_4_ served as a contrast agent for magnetic resonance imaging [77], and the SiO_2_ layer enabled further surface functionalization and increased the biocompatibility of the nanoplatform, while the TiO_2_ layer acted as a photosensitizer used in PDT [76].

Another type of nanostructure used was a Janus-type hybrid nanoparticle used by Ma et al. [78]. Janus nanoparticles are a special type of structure characterized by an asymmetrical surface structure, where the two hemispheres of the particle have different chemical or physical properties [79], which provides desirable properties that are not available in symmetric or homogeneous nanostructures [80,81]. The Janus core of the nanoparticles designed by Ma et al. consisted of a silver sulfide (Ag_2_S) segment, acting as an active therapeutic component capable of generating reactive oxygen species during PDT, and a gold segment, which was responsible for the formation of surface hot spots (SERS hotspots) necessary to enhance the Raman scattering signal of molecular probes used to monitor ATP levels and also enabled conjugation with a peptide that directs the nanoparticles to mitochondria. This core was further functionalized by coupling a DNA chain terminated with a thiol group at the 5′ position, which allowed the formation of an Au–S bond and the attachment of 5 nm diameter gold nanoparticles coated with p-mercaptobenzonitrile at the 3′ end to act as probes for enhanced Raman spectroscopy [78]. Raman scattering occurs as a result of the inelastic interaction of a photon with a molecule, leading to a shift in the frequency of light by a value corresponding to the vibrations of chemical bonds, thus obtaining a characteristic molecular “fingerprint” of the tested compound [82]. However, the native intensity of the Raman signal is low, which significantly limits its direct use in biotechnological systems due to strong background noise and a poor signal-to-noise ratio [83]. To overcome these difficulties, the surface-enhanced Raman scattering technique uses plasmonic nanostructures (usually based on gold or silver) to amplify the Raman signal by up to several orders of magnitude [83,84]. This enables the detection of single molecules or very low concentrations of analytes in cells, opening up new perspectives in biological and medical analysis [85]. In their work, Ma et al. confirmed that the use of Janus nanoparticles can combine the function of PDT with precise in situ monitoring of ATP levels in cancer cell mitochondria using surface-enhanced Raman scattering [78].

Metal–organic frameworks (MOFs) also represent a promising class of nanostructures explored for PDT agent development in cervical cancer [86]. MOFs are porous materials formed by the self-assembly of metal ions or metal clusters with organic bridging ligands. They are characterized by unique properties such as tunable chemical composition, designable and controllable structure, high porosity, ease of functionalization, and good biocompatibility [87]. Thanks to their ability to efficiently perform carrier functions, MOFs have gained significant recognition and found application in PDT [88]. Gao et al. used the MOF structure in their work to construct an IMF nanocomposite. Its core is a modified metal–organic framework UiO-66-(COOH)_2_ (UMOF), built of hexanuclear clusters Zr_6_O_4_(OH)_4_(CO_2_)_12_, connected by a 1,2,4,5-benzenetetracarboxylic ligand (H_4_btec). A thin amorphous layer of FeOOH [86] was deposited on the surface of the UMOF. Coordinated Fe^3+^ ions exhibit catalase-like activity [89] and are capable of decomposing hydrogen peroxide (H_2_O_2_), which allows for the local generation of molecular oxygen. In a tumor environment, where H_2_O_2_ concentrations can range from several to several dozen µM—in contrast to nanomolar levels in healthy tissues [90]—a strategy based on local O_2_ production is a promising approach to counteract hypoxia and improve the effectiveness of PDT [91]. In the work of Gao et al., the FeOOH layer acted as a catalase-like active center, which was achieved through strong coordination of Fe^3+^ ions with UMOF carboxyl groups. In vitro studies in HeLa cells confirmed this concept and showed that IMF nanoparticles effectively generated molecular oxygen under normoxic (21% O_2_) and hypoxic (2% O_2_) conditions, leading to a reduction in the level of hypoxia-induced HIF-1α. Furthermore, it was shown that immobilization of an amorphous FeOOH layer on a UMOF support significantly increased the efficiency of H_2_O_2_ decomposition compared to free FeOOH, indicating a synergistic enhancement of catalase-like activity resulting from the coordination of Fe^3+^ ions with the carboxyl groups of the UMOF structure [86]. Yang et al. also used an MOF-based nanostructure to construct PMOF@AuNP/hairpin. PMOF@AuNP/hairpin is a hybrid nanoparticle on the surface of which gold nanoparticles were grown in situ. Similar to the work of Gao et al., MOF provided catalase-like properties, allowing the decomposition of H_2_O_2_ into molecular oxygen. The structure was then functionalized with Cy5/BHQ2-labeled hairpin DNA probes for selective miRNA detection. To improve biological stability and accumulation in the tumor, the nanoparticle was additionally encapsulated in a zeolite imidazolane framework type 8 (ZIF-8) and coated with a PEG layer [92].

A supramolecular nanostructure studied in the context of PDT for cervical cancer was the TAT-InTPP nanoparticle developed by Yang et al., composed exclusively of indoporphyrin (InTPP) molecules that self-assembled into various morphologies—including nanorods, nanospheres, nanoplates, and nanoparticles—via an oil-in-water microemulsion process. Among these, nanorods were selected for in vivo studies due to their highest singlet oxygen generation efficiency [93]. Porphyrins, natural macrocycles with a strongly coupled π-electron system [94], form stable supramolecular assemblies due to their flat and their branched structure and ability to coordinate metal ions [95,96]. The incorporation of a heavy In^3+^ ion into the center of the InTPP macrocycle increases the efficiency of intersystem crossing and prolongs the lifetime of triplet states, which promotes more efficient singlet oxygen generation [97,98]. J-type aggregates play a special role here, with InTPP molecules arranged head to tail, leading to a shift in absorption toward longer wavelengths and greater electron delocalization [99,100,101] and consequently to improved photodynamic properties [102,103,104]. Controlled nanowire formation was achieved using CTAB, whose cationic nature [105] stabilizes the positive surface charge of molecules in an aqueous environment and enables the growth of uniform rods with adjustable length and diameter [93].

A polymeric colloidal nanoparticle system applied in PDT for cervical cancer was proposed by Gao et al. This nanoparticle consists of a copolymer carrier composed of a mixture of two block polymers: mPEG-PLGA and PLGA-b-PEG-TPP, a polymer modified at the end of the hydrophilic chain with a triphenylphosphine (TPP) group, responsible for targeting the mitochondria. Both units together form an amphiphilic polymer system that spontaneously self-assembles into spherical colloidal nanoparticles in a nanoprecipitation process. The interior of the nanoparticle acts as a retention phase for two lipophilic compounds: atovaquone and IR780, a hydrophobic photosensitizer. The outer layer is composed of hydrophilic PEG segments, which ensure colloidal stability and prolonged circulation time [106].

Nanocarriers based on mesoporous silicon oxide represent another class of nanostructures explored in PDT for cervical cancer (Table 2). Chen et al. developed spherical, double-layered, hollow mesoporous silica nanoparticles [107]. Multilayer mesoporous spheres with hollow structure are characterized by increased load capacity, making them suitable carriers for photosensitizers and other bioactive molecules [108,109]. The core of the nanocarrier is a double-layered hollow silica structure connected by organic bridges, the space between the inner and outer shells forming a closed cavity capable of effectively encapsulating the load [109,110]. Chen et al. loaded these nanoparticles with the photosensitizer zinc (II) phthalocyanine (ZnPc) and the liquid perfluoropentane, obtaining the DSi@Z/P system [107]. Perfluoropentane, with a phase transition temperature of approximately 28 °C [111], was introduced into the nanoparticles by vacuum infusion. Under physiological conditions, this compound undergoes a phase transition to a gaseous phase, leading to the formation of microbubbles, which increase local acoustic impedance and thus improve contrast in in vivo ultrasound imaging [112]. The authors demonstrated that DSi@Z/P can serve as an effective contrast agent in ultrasound imaging. Furthermore, in a mouse model of a subcutaneous HeLa tumor, this nanoplatform showed better therapeutic efficacy and higher imaging quality compared to free ZnPc and monolayer nanoparticles [107]. Ye et al. designed an advanced PMnSAGMSNs-V@Ce6 nanosystem based on a mesoporous silica carrier enriched with atomically dispersed transition metal centers—manganese and gadolinium—and oxygen vacancies obtained by the hydrothermal method [113]. Oxygen vacancies are a type of structural defect in the crystal lattice of metal oxides, consisting of the absence of an oxygen atom in a lattice node that should normally be occupied. The introduction of oxygen vacancies is an effective strategy to increase the photocatalytic activity of nanomaterials. These defects act as traps for charge carriers, limiting their recombination and broadening the range of light absorption, e.g., through plasmonic effects. In addition, they promote the activation of molecules such as O_2_ and H_2_O_2_, weakening their bonds and facilitating catalytic reactions, including Fenton-like reactions [113,114,115,116]. Hydrothermally generated oxygen vacancies also allow the capture of metal ions and the creation of atomic sites, which further increases the efficiency of hydroxyl radical generation [117]. Doping silica with metal ions enabled its excitation with a 650 nm wavelength laser to produce electron-hole pairs, thus facilitating the generation of reactive oxygen species. Furthermore, the presence of metals ensured the generation of superoxide and hydroxyl radicals, resulted in the depletion of glutathione, and provided tumor-specific contrast agents for magnetic resonance imaging [113].

## 3. Photosensitizers Used in Nanoplatforms for PDT of Cervical Cancer

The design of nanoplatforms intended for PDT of cervical cancer uses both well-known and extensively tested photosensitizers, as well as newly synthesized compounds with improved photophysical and biological properties. The integration of these photosensitizers with nanostructured carriers allows for increased solubility, stability, precision of delivery to cancer cells, and efficiency of reactive oxygen species generation. The photosensitizers most commonly used in nanoparticles for PDT of cervical cancer were porphyrins and their derivatives. Porphyrins are a group of organic macrocyclic compounds consisting of four pyrrole rings forming a porphyrin system. They are natural macrocycles with an extensive conjugated π electron system [94], which, due to their flat structure and ability to coordinate metal ions, can form stable supramolecular assemblies. Porphyrins strongly absorb light in the 400–450 nm and 500–700 nm ranges [95,96]. Tetrakis(4-carboxyphenyl)porphyrin (TCPP) has been used in nanosystems PMOF@AuNP/hairpin [92], TPP-UCNP@MOF-Pt [54], and Fc-CA-PCN-HA [59] nanosystems. TCPP is characterized by a broad absorption band in the range of ~415–420 nm and 515–650 nm [118]. TCPP has the ability to self-assemble with metal ions in the MOF structure, which ensures a high photosensitizer loading capacity while preventing aggregation and fluorescence quenching, significantly increasing the effectiveness of therapy [119]. Importantly, TPP-UCNPs@MOF-Pt uses UCNPs, which enable the use of NIR [54]. The TAT-InTPP nanoplatform design uses InTPP, a porphyrin derivative with an In^3+^ ion at the center of the macrocycle. The presence of a heavy ion promotes efficient transitions and prolongs the lifetime of triplet states, resulting in more efficient singlet oxygen generation [97,98]. Another commonly used photosensitizer was ZnPc. Phthalocyanines are aromatic heterocycles composed of four isoindole rings, which belong to the group of second-generation photosensitizers. They are characterized by a maximum absorption wavelength greater than 670 nm and a high molar extinction coefficient [120], which translates into more efficient singlet oxygen generation [121]. Phthalocyanines exhibit low or even no absorption in the 400–600 nm range, where the intensity of sunlight is highest. This significantly reduces the risk of photosensitivity of the skin caused by daylight [122]. The maximum absorption of ZnPc occurs in the Q band around 670 nm, which places it in the red-light range but does not include NIR [123]. To enable the activation of ZnPc by light with a longer wavelength and better tissue penetration, UCNP was used on the UCN@mSiO_2_@ZnPc@L-Arg, and UM-RZ nanoplatforms [51,52] were used in the UCN@mSiO_2_@ZnPc@L-Arg and UM-RZ nanoplatforms. However, this approach was not used in the case of DSi@Z/P [107]. Another second-generation photosensitizer used was Chlorin e6, used in the PMnSAGMSNs-V@Ce6 nanosystem [113]. Chlorin e6 is a semisynthetic photosensitizer from the chlorin family widely used in PDT. It exhibits maximum light absorption at 400 and 665 nm and fluorescence, allowing its activation by red light [124]. However, in the case of the PMnSAGMSNs-V@Ce6 nanocomposite, UCNP was not used [113]. UCNP was used in the UCNP@Glu-DMMA-PDT nanoparticle, where TiO_2_ was used as a photosensitizer, which requires UV light with poor penetration of deep tissue for activation [76]. Another photosensitizer used was IR780, which was used in the design of the TNP/IA system [106]. IR-780 is a heptamethylene cyanine dye with a maximum absorption wavelength of 780 nm and a high molar extinction coefficient, operating in the NIR range. It is distinguished by strong fluorescence in the 790–826 nm range and high quantum yield, which makes it not only an effective photosensitizer but also a useful tool in biological imaging [124,125]. This property has been exploited in TNPs/IA, where the dye molecule is precisely delivered to mitochondria and its autofluorescence enables real-time monitoring of nanoparticle biodistribution [106]. Similar to IR780, indocyanine green is a dye operating in the NIR range, exhibiting maximum absorption at approximately 785 nm in aqueous solution, which shifts to approximately 805 nm after binding to plasma proteins [126,127]. This cyanine photosensitizer has been used in the design of an IMF nanocomposite [86]. Importantly, indocyanine green has already been approved by the FDA [128]. Some of the studies used completely newly synthesized photosensitizers. Nsubuga et al. synthesized maleimide-TCI. Maleimide-TCI is a newly designed compound of the class of thiochromenocarbazole imides, which is a modified version of the previously described TCI chromophore [129,130]. The compound, modified with a maleimide group, enables permanent covalent attachment to UCNP nanoparticles via a thiol-maleimide reaction. This system enables efficient energy transfer (FRET) between Tm^3+^ ions in UCNPs and the photosensitizer molecule. Maleimide TCI exhibits strong absorption at 405 and 485 nm, emission at 526 nm, and very high singlet oxygen generation efficiency, surpassing other known heavy metal-free photosensitizers such as BODIPY or thio-coumarin. In the study described, maleimide-TCI was attached to the surface of UCNP modified with mercaptopropionic acid, and then the whole was covered with a phospholipid layer to increase stability and biocompatibility [53]. Ling et al. synthesized a BODIPY derivative and used it in the UCNP@SiO_2_-Bodipy@FFYp nanoparticle [75]. BODIPY dyes are fluorescent compounds with high stability and significant chemical modification capabilities, which are increasingly used as photosensitizers in PDT. Their limitation is the low efficiency of singlet oxygen generation, resulting from the limited transition to the triplet state [131,132]. In the described work, BODIPY was modified by coupling with a phenyl-alkyne group to form Bodipy-I, which improved its photophysical properties and enabled its deposition in a silicon oxide coating. In the UCNP@SiO_2_-Bodipy@FFYp nanoparticle, the FRET mechanism was used, where the UCNP absorb NIR radiation (980 nm) and Bodipy-I generates singlet oxygen [75]. Zhao et al. synthesized MEO-TTMN, which belongs to the class of compounds exhibiting AIE. In its molecular state, MEO-TTMN absorbs light at 506 nm and emits light at 703 nm; after encapsulation in silica nanoparticles, the maximum emission shifts to 675 nm. This compound is characterized by high specificity, stability, and therapeutic efficacy. It was used in the construction of the MT@SiO_2_-MP NPs nanoplatform [57]. In the study by Liu et al., modified graphitic carbon nitride—C_3_N_4_-RP—was used in the construction of the C_3_N_4_-RP@RBCm nanoplatform [58]. Graphitic carbon nitride (g-C_3_N_4_) is a semiconductor material composed of carbon and nitrogen, characterized by high chemical stability and a flexible electronic structure. Its use in PDT is limited due to its narrow absorption range (mainly UV) and rapid recombination of photogenerated charge carriers. A modified version—C_3_N_4_-RP—obtained by doping with red phosphorus, exhibits absorption of red light (660 nm), generates 2.3 times more singlet oxygen compared to unmodified C_3_N_4_, and is characterized by lower phototoxicity and greater efficacy in deeper tissues [58,133]. In another study described, a classic photosensitizer in the form of a single molecule was not used, but JMDA nanoparticles were used as a complex functional photosensitizer in the form of a complete nanoplatform [78].

## 4. Strategies for Delivering Nanosystems for Cervical Cancer

The precise delivery of drugs to cancer cells remains one of the most important challenges in modern cancer therapy. Currently, systemic methods of transporting nanosystems are based mainly on two mechanisms: passive and active. Technological advances have also enabled the development of nanomaterials that respond to specific stimuli present in the tumor microenvironment, allowing the use of passive and active mechanisms [134,135].

In the passive targeting mechanism, nanosystems without target ligands accumulate in the interstitial space of the tumor as a result of the enhanced permeability and retention (EPR) phenomenon. This effect results from abnormal blood vessel structure and impaired lymphatic drainage within the tumor [136,137]. The EPR mechanism is particularly characteristic of solid tumors, which promotes the accumulation of nanostructures with a size of 20–500 nm [138,139]. Despite promising results in preclinical studies, the clinical application of the EPR effect remains limited. Many nanoplatforms do not show reproducible therapeutic efficacy in patients [140]. The main problem is the significant heterogeneity of this effect, observed both between different patients and tumor types, as well as within individual tumors. It has been observed that the heterogeneity of the EPR effect is lower in xenograft models than in human tumors transplanted into mice [141]. These differences result from factors such as vascular density, the number and size of intercellular gaps, the degree of thrombogenesis, interstitial pressure, and the efficiency of lymphatic drainage [141,142,143]. The EPR effect is usually more pronounced in small, fast-growing tumors typical of laboratory conditions [142]. In rodents, tumors reach a diameter of about 1 cm within 2 to 3 weeks, leading to the development of pathologically permeable vessels. On the contrary, human tumors develop over many years, and their vasculature typically exhibits lower permeability [144]. Therefore, the validity of using EPR as the main mechanism for the delivery of drugs to tumors is increasingly being questioned, and researchers are looking for alternative strategies to enable more effective delivery of nanoparticles to tumors [142,145]. However, many nanoparticles described in the literature, as well as those presented in this article [51,58,92,106,113], are still based on the passive EPR effect mechanism as the primary means of transport to the tumor, which may limit their translation into clinical practice. In some cases, these nanosystems have additionally been functionalized with organellotropic ligands, enabling their targeting to mitochondria. Targeting photosensitizers to mitochondria in PDT has significant therapeutic benefits. One of the main mechanisms of the cytotoxic effect is the induction of cytochrome c release from the mitochondrial intermembrane space, which is a key signal initiating the apoptosis cascade [146]. Therapeutic strategies focusing on mitochondria have shown higher efficacy compared to non-targeted anticancer approaches. In particular, in PDT, mitochondriotropic photosensitizers play an important role in overcoming barriers associated with tumor hypoxia, which translates into increased therapeutic efficacy [147]. The targeting of nanoplatforms to mitochondria was achieved both by Gao et al. [106], who incorporated TPP [148,149] into the structure of the PEG-PLGA copolymer [106], and by Chen et al., who functionalized the TPP-UCNPs@MOF-Pt nanoplatform with the TPP group [54]. This approach enables the selective accumulation of nanosystems in mitochondria, taking advantage of their negative membrane potential [150,151] and the lipophilic-hydrophilic nature of TPP. The advantages of TPP-based mitochondrial targeting over other approaches to the delivery of small mitochondrial molecules include the stability of the TPP group in biological systems, relatively simple synthesis and purification, low chemical reactivity with cellular components, and no light absorption or fluorescence in the visible or NIR spectrum [152]. Targeting TNPs/IA nanoparticles to mitochondria is particularly desirable due to the presence of atovaquone [106], a clinically approved antimalarial drug [153]. Atovaquone inhibits the activity of the cytochrome bc_1_ complex in the mitochondrial respiratory chain, leading to inhibition of oxidative phosphorylation [154,155,156], which is the main pathway for ATP production in cancer cells [157]. As a result, tumor hypoxia is reduced, and the effectiveness of PDT is increased [158]. The authors confirmed the ability of the TPP groups to effectively target nanoparticles to mitochondria. Atovaquone has also been shown to effectively inhibit mitochondrial cellular respiration, leading to a decrease in oxygen consumption and ATP production. This effect was strongest in the group of TPP-functionalized nanoparticles, highlighting the important role of targeted delivery of atovaquone to mitochondria [106]. Chen et al. also confirmed the effectiveness of TPP as a mitochondriotropic ligand by functionalizing their TPP-UCNPs@MOF-Pt nanoparticle, which enabled its precise targeting to mitochondria. In comparison, nanoparticles without the TPP group showed significantly weaker mitochondrial co-localization, proving that the presence of TPP significantly increases the specificity of delivery to mitochondria [54].

Although the EPR effect enables passive accumulation of nanoparticles within the tumor, the limited effectiveness of internalization into cancer cells is a significant barrier to effective therapy. In response to this challenge, strategies that combine EPR with mechanisms that facilitate the penetration of nanoparticles through the cell membrane are being developed. To this end, Yang et al. coated the TAT-InTPP nanorods with a covalently conjugated TAT peptide with DSPE-PEG-NHS [93]. DSPE-PEG is a widely used phospholipid–polymer conjugate in drug delivery applications. It is a biocompatible, biodegradable, and amphiphilic material that can also be functionalized with various biomolecules to achieve specific functions [159]. The TAT peptide is an HIV-1 protein that is an essential viral regulatory factor that plays a key role in viral replication [160]. TAT penetrates cells very quickly and independently of receptors, most likely through direct electrostatic interaction with cell membrane components. Internalization does not require receptors or classical endocytosis, and its efficiency can be reduced by competition with polyanions [161]. A study by Yang et al. confirmed the validity of the concept of using the TAT peptide. Coupling nanorods with the TAT peptide significantly increases their uptake and accumulation in cancer cells compared to the control group without the TAT peptide [93]. Another approach was taken by Wang et al., who developed the UM-RZ nanostructure, in which the targeted transport, as described above, is based on the generation of NO from Roussin black salt. The locally produced gas not only increases the mobility of the nanoparticles in the tumor environment but also induces stress in cell membranes, facilitating their endocytosis and significantly improving penetration within the tumor, compared to the control group without Roussin salt. A key element of this strategy is the multilayer structure of UCNP (NaErF_4_:Tm@NaYF_4_@NaYbF_4_:Tm@NaYF_4_), which enables light emission at different wavelengths depending on the NIR source used. Sequential excitation with 980 nm light leads to the release of NO and the activation of nanoparticle movement toward cancer cells, while 808 nm light activates ZnPc, resulting in ROS generation. This approach integrates active transport with controlled therapeutic activation, maximizing the delivery of nanostructures to tumor cells [52].

Although strategies such as the use of cell-penetrating peptides or the local release of gases such as NO effectively support the penetration of nanoparticles into the tumor and their internalization into cells, they do not ensure selectivity with respect to cancer cells. Due to the limitations of the EPR mechanism, there is growing interest in active targeting. This involves functionalizing the surface of nanosystems with molecular ligands, such as antibodies, peptides, or small molecules that selectively bind to receptors overexpressed on the surface of cancer cells. This process often results in the internalization of the nanoplatform through receptor-dependent endocytosis [162,163]. Active targeting increases the selectivity of drug delivery and may improve its efficacy [164,165]. However, this approach also has limitations. These include heterogeneous receptor expression in different tumor cell populations and the so-called binding site barrier, which involves a strong and early binding of nanoplatforms in peripheral areas of the tumor, preventing their further diffusion into tumor tissue [144,166,167]. The hyaluronan coating used by Bai et al. [59] gives nanoparticles targeted delivery properties to cancer cells through interaction with the CD44 receptor [168]. CD44 expression is significantly elevated in tissues from cervical cancer compared to corresponding healthy tissues [169], and its level correlates with the increased aggressiveness of this cancer [170]. This makes hyaluronan a particularly attractive ligand in molecular targeting strategies. Bai et al. confirmed the validity of using hyaluronan as a targeting agent for cervical cancer cells by demonstrating the effective endocytosis of Fc-CA-PCN-HA nanoparticles by cancer cells. At the same time, a significantly lower level of uptake was observed in healthy LO2 cells, indicating the selectivity of nanoparticles towards cancer cells [59].

**Figure 1:**
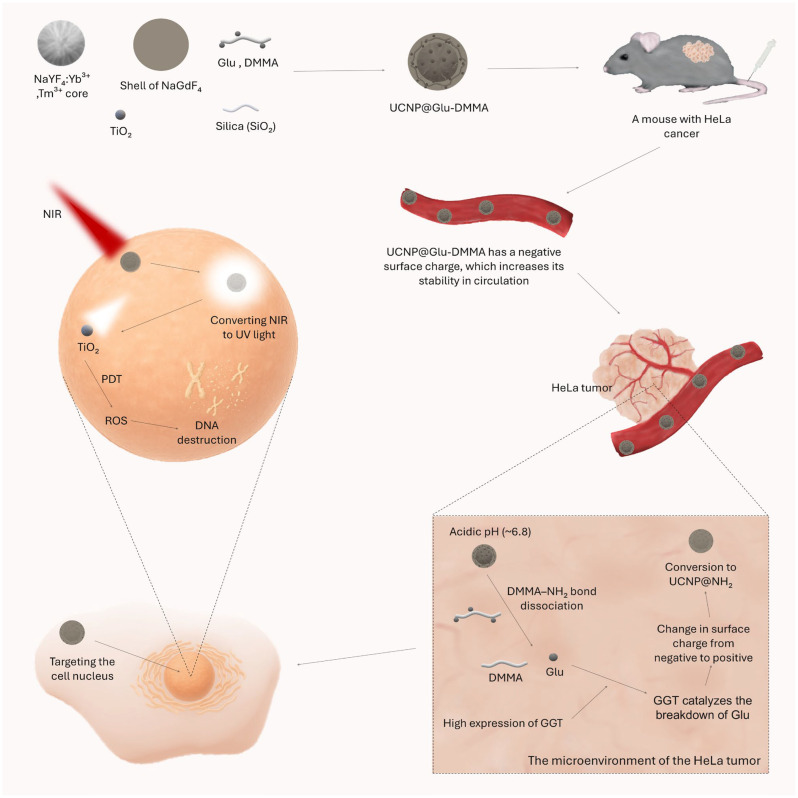
The figure shows the structure and mechanism of action of the UCNP@Glu-DMMA nanoplatform. The nanoparticles consist of a NaYF_4_:Yb^3+^, Tm^3+^ core, a NaGdF_4_ shell, and a silica (SiO_2_) coating covered with a layer of titanium oxide (TiO_2_). The surface of the nanoparticles has been functionalized with glutamic acid (Glu) and dimethyl maleate (DMMA) molecules, which gives them a negative surface charge. Thanks to this charge, UCNP@Glu-DMMA exhibit high stability in the bloodstream, do not aggregate, and are not prematurely captured by the reticuloendothelial system. Upon reaching the tumor microenvironment (TME), characterized by a slightly acidic pH (~6.8) and overexpression of the enzyme γ-glutamyl transpeptidase (GGT), a cascade reaction is triggered. The acidic pH causes the DMMA–NH_2_ bond to cleave, and GGT catalyzes the breakdown of Glu. As a result of these processes, the surface charge changes from negative to positive, leading to the conversion of UCNP@Glu-DMMA to UCNP@NH_2_. This change promotes effective uptake by cancer cells and the penetration of nanoparticles into their nuclei. When irradiated with near-infrared (NIR) light, the nanoparticles emit UV light, which activates the TiO_2_ coating to generate reactive oxygen species (ROS). The ROS produced damage DNA, leading to the death of cancer cells as a result of photodynamic therapy (PDT) [76].

An alternative approach is to target the characteristics of the tumor microenvironment, such as acidic pH, the presence of specific enzymes, or hypoxia [171]. The UCNP@Glu-DMMA nanoparticle designed by Ling et al. uses a strategy that increases both the specificity of nanoparticle targeting to cancer cells and their ability to penetrate the cell nucleus. The targeting of UCNP@Glu-DMMA is based on a two-step mechanism. The surface of the nanoparticle was functionalized with glutamic acid [76], which has a high affinity for γ-glutamyl transpeptidase (GGT), an enzyme that is overexpressed in actively metabolizing cancer cells [172], including HeLa cells [76]. GGT catalyzes glutathione breakdown and generates positively charged ions that initiate transcytosis [173]. Furthermore, the enzymatic release of free amino groups promotes the transport of nanoparticles to the cell nucleus [174], increasing local ROS production in the immediate vicinity of DNA, which maximizes cytotoxicity of PDT. To prevent premature uptake by healthy tissues, the primary-NH_2_ groups were masked with a DMMA (β-carboxylic amide) molecule [76], giving the nanocomposite a negative surface charge. This solution increases stability in circulation and reduces plasma protein adsorption and non-targeted endocytosis [175,176]. In the acidic microenvironment of the tumor [177], protonation and hydrolysis of DMMA amide bonds occur [175], resulting in glutamic acid and reversal of surface charge, enabling selective uptake by tumor cells in a “lock and key” mechanism. Research by Ling et al. confirmed the effectiveness of this concept. Under acidic pH conditions (6.8), characteristic of the tumor microenvironment, intense accumulation of UCNP@Glu-DMMA nanoparticles was observed in HeLa cells in contrast to the much weaker accumulation observed in HUVEC cells under physiological pH conditions (7.4) and in HeLa cells subjected to prior GGT inhibition. Importantly, targeting selectivity was also confirmed in vivo—UCNP@Glu-DMMA showed significantly higher accumulation in HeLa tumors compared to other organs (except for the liver and spleen). Administration of the GGT inhibitor led to a significant decrease in nanoparticle accumulation in the tumor, confirming the dependence of the delivery mechanism on the activity of this enzyme. Furthermore, the presence of UCNP@Glu-DMMA inside the cell nuclei of HeLa cells was confirmed, indicating effective penetration of both the cell membrane and the nuclear membrane [76]. The mechanism of action of the UCNP@Glu-DMMA nanoparticle is shown in Figure 1.

Yang et al. also applied the approach using the acidic microenvironment of the tumor, who encapsulated PMOF@AuNP/hairpin nanoparticles in a layer of ZIF-8 and PEG [92]. ZIF-8 is a non-toxic biodegradable material that is sensitive to slightly acidic conditions (pH 5.0–6.0) as a result of the protonation effect. This structure not only effectively protects the active ingredients from degradation in vivo but also enables controlled, pH-dependent release of therapeutic molecules in the tumor microenvironment. This allows for their preferential accumulation in tumor tissue and a selective cytotoxic action [178]. The effectiveness of this approach has been experimentally confirmed—ZIF-8 has demonstrated the ability to selectively degrade under acidic conditions, allowing the release of functional components of the nanostructure within the tumor [178]. In the third study that used a tumor microenvironment targeting approach, Ling et al. used the FFYp peptide in their UCNP@SiO_2_-Bodipy@FFYp nanoparticle [75]. The FFYp peptide (Phe-Phe-Tyr-(PO(OH)_2_)-OH) is a short phosphorylated tripeptide designed as a substrate for alkaline phosphatase (ALP). In the presence of ALP, the enzyme catalyzes the dephosphorylation of FFYp, leading to the formation of the hydrophobic peptide FFY (Phe-Phe-Tyr-OH), which undergoes self-assembly [179]. The process of dephosphorylation and self-assembly promotes the internalization of nanoparticles and increases the effectiveness of PDT through the local accumulation of photosensitizer within the tumor [180]. In their study, Ling et al. confirmed that the FFYp peptide enables ALP-dependent dephosphorylation, leading to the induction of nanoparticle aggregation in cells overexpressing ALP and resulting in increased accumulation of nanoparticles in cancer cells, which translates into significantly higher PDT efficiency both in vitro and in vivo compared to nanocomposites without peptide modification [75]. However, it should be emphasized that the tumor microenvironment in humans differs significantly from that observed in animal models [181,182], which can hinder the effective use of these nanoplatforms in clinical settings.

One way to bypass the physiological barriers associated with systemic administration is to inject nanoparticles directly into the tumor, as used in the nanostructures UCNP-M-TCI (Nsubuga et al.) [53], MT@SiO_2_-MP (Zhao et al.) [57], JMDA (Ma et al.) [78], IMF (Gao et al.) [86], and DHMSN (Chen et al.) [107]. Although this method allows systemic barriers to be bypassed, it is usually characterized by a more heterogeneous distribution of nanomaterials within the tumor, which can limit its effectiveness compared to the more uniform distribution after intravenous administration [53]. In addition, in the case of nanosystems administered directly to the tumor, these nanosystems were additionally functionalized with organellotropic ligands. Zhao et al. used a strategy to target nanoparticles to lysosomes by functionalizing MT@SiO_2_-MP with morpholine groups [57]. Under physiological pH conditions, morpholine remains unprotonated and neutral, while in the acidic lysosomal environment, it undergoes protonation, which promotes the retention of nanoparticles within these organelles as a result of electrostatic interactions [183,184]. Lysosomes, which play an important role in endocytic pathways, cellular metabolism, and the regulation of tumor proliferation and resistance, are an attractive therapeutic target [185]. Their damage can induce immunogenic cell death [186], contributing to the activation of the immune response, especially in the context of PDT [187]. The study demonstrated an effective colocalization of functionalized nanoparticles with a lysosomal marker in HeLa cells, suggesting a high ability to target lysosomes. In addition, the stability of the MT@SiO_2_-MP structure in an acidic environment simulating lysosomal conditions (pH = 5.0) was confirmed [57]. Ma et al. used a synthetic peptide Mitochondria-Targeting Sequence (MLALLGWWWFFSRKKC) to modify the surface of JMDA nanoparticles for targeting to mitochondria. Due to the presence of an amphipathic α-helix and a positive net charge, this peptide exhibits selective affinity for mitochondria, exploiting its negative membrane potential. Mitochondrial targeting of JMDA is particularly justified due to its additional function: they enable real-time monitoring of ATP levels directly at the site of therapeutic action. This allows for dynamic evaluation of the effectiveness of therapy and the metabolic state of cancer cells during photodynamic induction of mitochondrial damage. The authors confirmed the ability of JMDA nanoparticles to selectively localize in mitochondria, making them a promising photosensitizer carrier and a tool for in situ monitoring of energy metabolism during PDT [78].

## 5. Other Strategies to Enhance the Effectiveness of PDT Using Nanosystems

**Figure 2:**
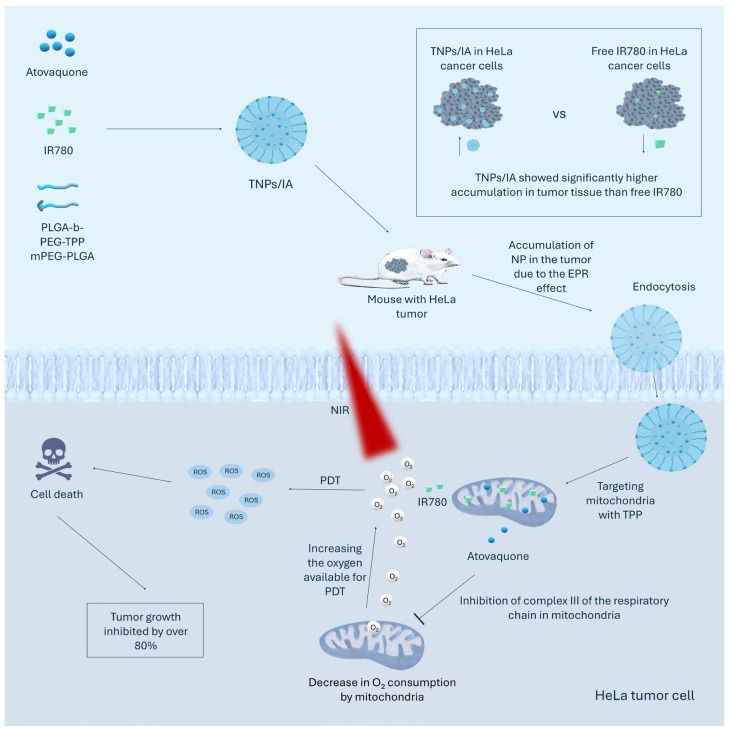
The figure shows the structure and mechanism of action of TNPs/IA nanoparticles. These nanoparticles (NPs) consist of the photosensitizer IR780, atovaquone, and the copolymers mPEG-PLGA and PLGA-b-PEG-TPP. TNPs/IA have been shown to accumulate in tumor tissue much more efficiently than free IR780. After intravenous administration, these molecules preferentially accumulate in the tumor due to the EPR effect (enhanced permeability and retention) and are subsequently internalized by HeLa tumor cells through endocytosis. Due to the presence of triphenylphosphonium cation (TPP), TNPs/IAs are selectively targeted to mitochondria, taking advantage of their negative membrane potential. In mitochondria, atovaquone inhibits the activity of respiratory chain complex III, reducing oxygen consumption and thereby alleviating hypoxia and increasing the efficacy of photodynamic therapy (PDT). At the same time, IR780, activated by near-infrared (NIR) radiation, generates reactive oxygen species (ROS) that induce mitochondrial damage and cancer cell death. Application of PDT therapy with TNPs/IA led to inhibition of tumor growth by more than 80% in an in vivo model [106].

In addition to strategies focused on carrier structure, photosensitizer type, and delivery mechanisms, the efficacy of PDT can be further enhanced by the use of adjuvant components that interact with the tumor microenvironment, influence the production of reactive oxygen species, modify hypoxia levels, or generate additional cytotoxic effects. This chapter discusses examples of such approaches that integrate, among others, functional amino acids, inorganic enzymes, redox catalysts, mitochondrial inhibitors, and other bioactive components that enhance the effect of PDT in vitro and in vivo. Chen et al. proposed a strategy for using PtNPs as part of the TPP-UCNPs@MOF-Pt nanoparticle [54]. PtNPs, characterized by high stability and biocompatibility, have the ability to effectively catalyze the decomposition of H_2_O_2_ into oxygen in cancer cells. The incorporation of PtNPs into PDT systems is a promising approach to alleviate tumor hypoxia, which may significantly increase the effectiveness of PDT [188]. PtNPs are distinguished by their excellent catalytic activity, especially in redox reactions involving complex molecules [189]. Chen et al. confirmed the validity of using PtNPs in TPP-UCNPs@MOF-Pt. This nanoparticle effectively catalyzes the decomposition of H_2_O_2_, as demonstrated by a significant increase in oxygen concentration and the observation of visible gas bubbles. Under hypoxic conditions, nanoplatforms with PtNPs generated significantly higher amounts of singlet oxygen than nanoplatforms without PtNPs, indicating effective alleviation of hypoxia and improved ROS production. The effectiveness of PtNPs in reversing hypoxia was also confirmed in vivo in a mouse model with HeLa tumors, where TPP-UCNPs@MOF-Pt showed the highest therapeutic activity compared to nanoplatforms without PtNPs. Bai et al. proposed a strategy based on conjugates of ferrocene and cinnamaldehyde (CA), implemented in the Fc-CA-PCN-HA nanosystem [59]. Ferrocene is an organometallic compound with a characteristic “sandwich” structure in which an iron atom is sandwiched between two cyclopentadienyl rings. Ferrocene derivatives are distinguished by several beneficial chemical and physicochemical properties, such as high stability to air, temperature, and light, low toxicity, economical synthesis, the ability to undergo reversible redox reactions and ligand exchange, and catalytic activity [190]. CA is a bioactive compound derived from the natural cinnamon plant, which exhibits multiple anticancer mechanisms, including induction of apoptosis through caspase activation and mitochondrial dysfunction, inhibition of angiogenesis, and antiproliferative, anti-inflammatory, and antioxidant effects [191,192]. In addition, CA exhibits the ability to activate NADPH oxidase, leading to increased production of H_2_O_2_ [193]. The conjugate used in Fc-CA-PCN-HA acted as pH-sensitive Fenton agents, which are released into the acidic tumor microenvironment and catalyze the decomposition of elevated H_2_O_2_ concentrations into highly reactive hydroxyl radicals in the Fenton reaction. The oxygen produced in this process contributes to the alleviation of hypoxia in the tumor microenvironment. At the same time, CA released from the conjugate activates NADPH oxidase, increasing the production of H_2_O_2_, which is the fuel for the Fenton reaction. Studies conducted by Bai et al. confirmed the effectiveness of the proposed mechanism of action of the conjugate. An important aspect is the fact that nanosystems containing this conjugate showed the highest therapeutic efficacy both in vitro and in an in vivo mouse model [59] despite CA’s ability to act as a ROS scavenger [192]. Gao et al. used a strategy based on atovaquone in the TNPs/IA nanoparticle [106]. Atovaquone is a clinically approved antimalarial drug [153] that inhibits the function of cytochrome bc_1_ in the mitochondrial respiratory chain and thus, by blocking oxidative phosphorylation [154], the main pathway of ATP production in cancer cells [157], as well as alleviating tumor hypoxia and increasing the effectiveness of PDT [158]. In vitro studies in HeLa cell lines showed that TNPs/IA effectively inhibited oxygen consumption by mitochondria. In cells treated with TNPs/IA, ATP levels dropped to only 37.8% of the initial value, which is convincing evidence of blocking of the respiratory chain in mitochondria. Furthermore, under hypoxic conditions, TNPs/IA generated the highest ROS concentrations compared to control groups, which was due to the simultaneous inhibition of OXPHOS and increased availability and precise localization of IR780 in mitochondria. Control groups without atovaquone (free IR780, NPs/I) showed significantly weaker activity in vitro and in vivo in the HeLa TNPs/IA tumor [106]. The mechanism of action of the TNPs/IA nanoparticles is shown in Figure 2.

**Figure 3:**
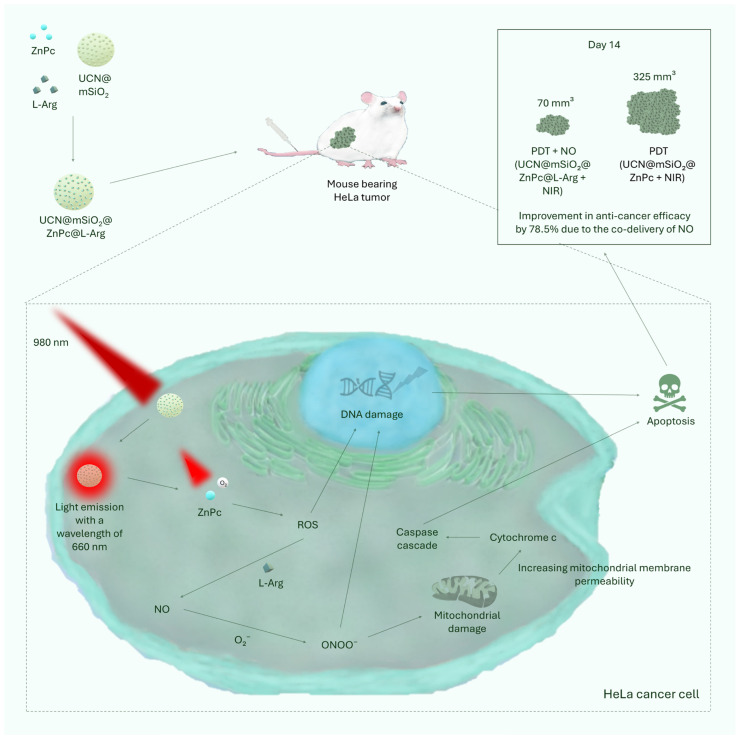
The figure shows the structure and mechanism of action of the UCN@mSiO_2_@ZnPc@L-Arg nanocomposite. It consists of three main components: the photosensitizer zinc phthalocyanine (ZnPc), the nitric oxide (NO) donor L-arginine (L-Arg), and upconverting nanoparticles (UCNPs) coated with mesoporous silica (mSiO_2_). When the nanocomposite is injected into tumors in mice with implanted HeLa cells and irradiated with near-infrared light (980 nm), the UCNP emits light at 660 nm. The resulting radiation activates ZnPc, which generates reactive oxygen species (ROS) in the presence of oxygen. ROS induce DNA damage and also react with L-arginine, leading to the formation of NO. NO then reacts with superoxide anions (O_2_^−^) to form peroxynitrite anions (ONOO^−^), compounds with strong cytotoxic potential. ONOO^−^ damages mitochondria, increases the permeability of their membranes, and causes the release of cytochrome c and activation of caspases, which ultimately induces apoptosis of cancer cells. In vivo studies have shown significant efficacy of PDT + NO combination therapy (UCN@mSiO_2_@ZnPc@L-Arg + NIR). After 14 days, the tumor volume in the group treated with PDT alone (UCN@mSiO_2_@ZnPc + NIR) was 325 mm^3^, while it had decreased to 70 mm^3^ in the group with PDT + NO therapy—an improvement in treatment efficacy of approximately 78.5% [51].

Lin et al. used a strategy based on the use of L-arginine in UCN@mSiO_2_@ZnPc@L-Arg [51]. L-arginine, a natural amino acid characterized by biocompatibility and low cost, serves as a precursor to NO in a reaction catalyzed by ROS generated during PDT [194,195]. NO has a dual effect on cancer cells [196,197]: at low concentrations (<100 nM), it promotes angiogenesis, proliferation, metastasis, and resistance to chemotherapy dependent on cGMP [198,199,200], while at high concentrations (>1 µM), it induces apoptosis through activation of the p53 pathway, release of cytochrome c, and protein modification [200,201,202,203,204]. In addition, the reaction of NO with ROS leads to the formation of peroxynitrite anions with strong cytotoxic properties [205]. Lin et al. confirmed the validity of the concept of using L-arginine as a component that enhances the effectiveness of PDT by inducing NO production. In the designed UCN@mSiO_2_@ZnPc@L-Arg nanocomposite, under the influence of near-infrared light (980 nm), L-arginine reacted with ROS generated by the ZnPc photosensitizer, leading to controlled, localized release of NO and, to a lesser extent, peroxynitrite. In vitro, nanocomposites with L-arginine showed significantly higher cytotoxic efficacy against HeLa cells than their L-arginine-free counterparts. Additionally, in the in vivo model (mice with implanted HeLa cells), the group treated with UCN@mSiO_2_@ZnPc@L-Arg-PDT showed the strongest inhibition of tumor growth compared to all control groups, both with PDT alone and with nanoparticles containing only L-arginine [51]. The mechanism of action of the UCN@mSiO_2_@ZnPc@L-Arg nanocomposite is shown in Figure 3.

## 6. Analysis of the Effectiveness of Nanosystems in PDT of Cervical Cancer

An analysis of the results of research on the use of nanosystems in PDT for cervical cancer showed that the greatest therapeutic efficacy was achieved in systems that targeted hypoxia modulation in the tumor microenvironment. The best results were achieved by the nanosystem designed by Gao et al. (IMF), which, after a single administration, led to complete regression of two of the three tumors and a marked reduction in the third tumor in just ten days. This efficacy was achieved despite the lack of a classical targeting system, suggesting that the properties of the nanostructure itself played a key role. IMF was designed as a MOF-based platform with catalase-like properties, capable of self-generating oxygen and enhancing the effect of photodynamic therapy [86]. Equally impressive results were achieved in a study by Gao et al. using the TNP/IA nanosystem. After a single injection, almost complete tumor regression was observed—its volume decreased from 100 mm^3^ to just 10 mm^3^ within 14 days, which is the best quantitative result among all the studies analyzed. This effectiveness was due to the mitochondrial targeting of the IR780 photosensitizer and the use of atovaquone, an oxidative phosphorylation inhibitor that reduces the oxygen consumption of cancer cells and thus improves the effectiveness of PDT under hypoxic conditions [106]. These results confirm the important role of strategies that improve oxygen conditions within the tumor to increase the effectiveness of PDT. In contrast, nanoplatforms that did not contain targeting components or additional mechanisms to enhance the photodynamic effect showed the lowest efficacy, emphasizing the importance of a properly designed nanocarrier structure. The UCNP-M-TCI nanosystem developed by Nsubuga et al. did not show statistically significant inhibition of tumor growth after a single administration. This nanosystem did not use targeting strategies or mechanisms to enhance the effectiveness of PDT [53]. Such strategies were also not used in the DSi@Z/P nanosystem designed by Chen et al. It proved ineffective—14 days after a single administration, tumor growth from 700 to 800 mm^3^ to approximately 1840 mm^3^ was observed [107]. However, it should be noted that the presence of targeting or PDT intensification strategies did not always translate into therapeutic success. In several cases, the use of such solutions did not result in satisfactory effects, suggesting that the effectiveness of nanosystems results from the synergy of many factors, rather than a single design element. Importantly, the dose regimen, including frequent or multiple administrations, was not a decisive factor in the effectiveness of treatment. Equally good or poor results were obtained regardless of the intensity of therapy, which further confirms that the architecture of the nanoparticle and its bioactive properties are of key importance. A similar observation was made with regard to the route of administration: both the most effective [86] and the least effective [53] nanosystems were administered intraarterially, which indicates that the site of administration itself does not determine therapeutic efficacy and that the quality and functionality of the nanocarrier system used plays a decisive role. The efficacy of all the nanosystems analyzed is summarized in Table 3 in order from most to least effective, according to the results described in individual studies.

## 7. Safety Assessment of Nanosystems

The studies analyzed assessed the safety of nanomaterials by monitoring body weight, performing histological analyzes of major organs and, in some cases, conducting biochemical serum tests and hematological analyses. The results clearly indicate that the nanomaterials used exhibit minimal or negligible toxicity at the doses administered. In all studies, no significant changes in body weight were observed in mice during therapy, and histological images stained with hematoxylin and eosin of major organs such as the heart, liver, spleen, lungs, and kidneys showed no damage or inflammatory changes in any of the models. Biochemical and hematological blood tests confirmed that liver and kidney function parameters and hematological indices remained normal and did not differ significantly from the control groups [52,75,76,78,106,107]. Only in the study by Nsubuga et al., which investigated hybrid UCNP-M-TCI nanoparticles composed of an organic photosensitizer and upconverting nanoparticles, was there no in vivo assessment of systemic toxicity symptoms [53]. In summary, nanoplatforms did not show significant systemic toxicity in vivo, confirming their potential in PDT of tumors.

## 8. Conclusions

The data collected clearly indicate that the integration of nanotechnology with photodynamic therapy can significantly increase the effectiveness of cervical cancer treatment. The use of modern nanoparticles as photosensitizer carriers not only makes it possible to precisely deliver drugs to selected cellular organelles (e.g., mitochondria or lysosomes) but also offers the possibility of multifunctional modification of the particle surface from the addition of targeted ligands to elements controlling movement (“motor-cargo”) to structures imaging the therapeutic process in real time. As a result, nanocarriers can effectively overcome the tumor hypoxia barrier, improving the generation of reactive oxygen species and tackling the difficulties associated with the limited light penetration and low solubility of traditional photosensitizers. Despite the rapid development of research on nanoparticles in PDT for cervical cancer, most of the proposed systems do not achieve results that would justify the initiation of preclinical studies in large animal models, let alone clinical trials. Of the 16 studies analyzed, only one case achieved complete tumor regression, which is an exceptional result compared to other studies. Some nanoplatforms showed promising therapeutic effects, leading to a marked reduction in tumor volume or partial inhibition of its growth. However, a significant number of systems showed insufficient therapeutic effects, despite the use of advanced targeting or PDT enhancement strategies. This low percentage of highly effective nanoplatforms may explain why so few of them reach further stages of development. Many studies report complex nanosystem designs and declarative strategies to target or enhancing the PDT effect, but the actual therapeutic results are moderate. Therefore, it is necessary to identify the common characteristics of the most effective strategies and isolate those approaches that actually translate into improved therapeutic effects in vivo. Based on available data, it can be concluded that some strategies aimed at overcoming tumor hypoxia show exceptionally high therapeutic efficacy in PDT of cervical cancer, including the only case of complete tumor regression among all the studies analyzed. However, it should be emphasized that the effectiveness of these approaches varied: of the six nanoplatforms targeting hypoxia that were evaluated, only two were among the most effective, while the others achieved moderate or low results. Therefore, once optimally effective nanosystems have been developed, it will be crucial to conduct further phases of research, primarily extensive toxicokinetic testing, standardization of nanomaterial production, and well-designed clinical trials, to confirm the safety, biodistribution, and long-term efficacy of this innovative therapeutic strategy. Thus, although the concept of using nanotechnology in PDT for cervical cancer remains promising, its actual clinical value depends on further rigorous research to verify the efficacy and safety of the developed systems in the context of patient treatment.

## Figures and Tables

**Table 1:** Criteria for the inclusion and exclusion of overgrowth-retained articles.

Inclusion Criteria
Articles describing photodynamic therapy
Articles describing cancer therapy
Articles describing nanoparticles and nanocomposites
Articles published from 2023 to July 2025
**Exclusion criteria**
Articles describing photodynamic therapy combined with other forms of therapy (chemotherapy, radiation therapy, gene therapy, photothermal therapy, etc.)
Articles describing cancers other than cervical cancer
Articles other than original research papers
Articles in which the results of therapy were described only in vitro
Articles in a language other than English

**Table 2:** The table shows the type of nanostructure, the system name, the structure used, the photosensitizer used, and the reference.

Type	Structure Type	Name	Photosensitizer	Ref.
Nanoparticles	Core–shell	TPP-UCNPs@MOF-Pt	TCPP (porphyrin derivative)	[54]
		Fc-CA-PCN-HA	TCPP (porphyrin derivative)	[59]
		MT@SiO_2_-MP NPs	MEO-TTMN (AIE-type)	[57]
		UCNP-M-TCI	M-TCI (maleimide–TCI)	[53]
		UM-RZ	Zinc phthalocyanine	[52]
	Core–shell–shell	UCNP@SiO_2_-Bodipy@FFYp	Bodipy-I	[75]
	Core–shell–shell–shell	UCNP@Glu-DMMA	TiO_2_	[76]
	Janus	JMDA	No classic—the whole structure acts as a photosensitizer	[78]
	MOF	PMOF@AuNP/hairpin	TCPP (porphyrin derivative)	[92]
	Supramolecular	TAT-InTPP	InTPP (mesotetraphenylporphyrinium indium(III) hydrochloride)	[93]
	Polymer	TNPs/IA	IR-780	[106]
	Mesoporous silica	DSi@Z/P	Zinc phthalocyanine	[107]
Nanocomposites	Core–shell	C_3_N_4_-RP@RBCm	C_3_N_4_-RP (graphitic carbon nitride + red phosphorus)	[58]
		UCN@mSiO_2_@ZnPc@L-Arg	Zinc phthalocyanine	[51]
	MOF	IMF	Indocyanine green	[86]
	Mesoporous silica	PMnSAGMSNs-V@Ce6	Chlorin e6	[113]

**Table 3:** The table shows the author’s details, structure name, baseline tumor volume, therapeutic regimen used, treatment efficacy, and reference number.

Author	Name	Approximate Initial Tumor Volume	Therapeutic Regimen	Effectiveness	Ref.
Gao et al.	IMF	75–100 mm^3^	One-off	2 of the 3 tumors have completely disappeared; the third has shrunk significantly after 10 days	[86]
Gao et al.	TNPs/IA	100 mm^3^	One-off	~10 mm^3^ after 14 days	[106]
Liu et al.	C3N4-RP@RBCm	80 mm^3^	One-off	~35–45 mm^3^ after 14 days	[58]
Yang et al.	TAT-InTPP	100 mm^3^	3 injections—on days 0, 2, 4	~65 mm^3^ after 11 days	[93]
Ling et al.	UCNP@SiO_2_-Bodipy@FFYp	60 mm^3^	4 injections—on days 0, 1, 3, 5	~39 mm^3^ after 15 days	[75]
Chen et al.	TPP-UCNPs@MOF-Pt	100 mm3	One-off	~70 mm3 after 14 days	[54]
Lin et al.	UCN@mSiO_2_@ZnPc@L-Arg	80 mm3	6 injections—on days 0, 2, 4, 6, 8, 12	~70 mm^3^ after 14 days	[51]
Bai et al.	Fc-CA-PCN-HA	100 mm^3^	5 injections—on days 0, 4, 8, 12, 16	~100–120 mm^3^ after 20 days	[59]
Ling et al.	UCNP@Glu-DMMA	100 mm^3^	One-off	~130 mm^3^ after 16 days	[76]
Wang et al.	UM-RZ	100 mm3	One-off	~150 mm^3^ after 14 days	[52]
Ma et al.	JMDA	100 mm^3^	4 injections—no information on which days	~150 mm^3^ after 14 days	[78]
Zhao et al.	MT@SiO_2_-MP NPs	50 mm^3^	One-off	~75 mm^3^ after 15 days	[57]
Yang et al.	PMOF@AuNP/hairpin	100 mm^3^	One-off	~150–200 mm^3^ after 15 days	[92]
Ye et al.	PMnSAGMSNs-V@Ce6	70 mm^3^	One-off	~105–140 mm^3^ after 14 days	[113]
Chen et al.	DSi@Z/P	700–800 mm^3^	One-off	~1840 mm^3^ after 14 days	[107]
Nsubuga et al.	UCNP-M-TCI	100 mm3	One-off	No statistically significant inhibition of tumor growth	[53]
